# ACCESS TO LOCAL ESTABLISHMENTS AND NEIGHBORHOOD PERCEPTIONS: A FOCUS ON THE ROLE OF LOCAL BANKING

**DOI:** 10.1093/geroni/igad104.1927

**Published:** 2023-12-21

**Authors:** Alyssa Goldman, Megan Doherty Bea

**Affiliations:** Boston College, Boston, Massachusetts, United States; University of Wisconsin-Madison, Madison, Wisconsin, United States

## Abstract

As an increasing number of older adults are aging-in-place, individuals’ perceptions of their neighborhood environments have emerged as significant social-contextual issues for healthy aging. Prior studies have focused on how neighborhood characteristics shape perceptions of residential neighborhoods. Far less research has focused on the role of local banking, despite the intersections between local banking establishments and a host of key community social and economic resources, particularly for the older adult population. We use nationally representative data from older adults interviewed at Rounds 2 and 3 of the National Social Life, Health, and Aging Project (N=4,589) linked with the National Establishment Time Series database to examine neighborhood perceptions as a function of local banking establishment density. We find that older adults who live in areas with higher rates of banking establishments report significantly higher levels of neighborhood safety, even when adjusting for a host of individual and neighborhood characteristics. Importantly, other types of establishments also shape perceived neighborhood safety, however, the significance of certain associations only emerges when we adjust for the presence of local banking. We suggest that local banks may be a lynchpin in catalyzing the broader socioeconomic vitality of a place, supporting older residents’ favorable perceptions of neighborhood safety. We discuss the implications of our findings for inequality in aging in light of unequal access to local banking across social groups, potentially increasing the vulnerability of older adults who are less likely to adopt mobile banking solutions and who are more likely to be susceptible to financial exploitation.

